# Retraction: MicroRNA-26a reduces synovial inflammation and cartilage injury in osteoarthritis of knee joints through impairing the NF-κB signaling pathway

**DOI:** 10.1042/BSR-2018-2025_RET

**Published:** 2024-05-29

**Keywords:** MicroRNA-26a, NF-κB signaling pathway, osteoarthritis, Synovial inflammation

This Retraction follows an Expression of Concern relating to this article previously published by Portland Press. This article is being retracted from *Bioscience Reports* at the request of the first author Zhi Zhao following receipt of a notification from a reader, alerting the Editorial Office to duplications between the BMS-345541 panel of Figure 6D and the miR-26a mimics panel of Figure 3D, with vertical flip and colour transformation. The Editorial Office also have additional concerns surrounding this paper, specifically:

- Duplications of cells in Figure 4A/7A OA and NC panels, as well as duplications of cells in Figure 4C/7C OA and NC panels, Figure 4C miR-26a mimics panel and Figure 7C BMS-345541 panel with horizontal flip transformation, Figure 4C/7C OA and miR-26a inhibitors+BMS-345541 panels with horizontal flip transformation, and Figure 7C NC and miR-26a inhibitors+BMS-345541 panels with horizontal flip transformation
- Duplications in the two Western blot bands for Figure 5A OA and NC P-IκBα groups with Figure 5C OA NC P-IκBα groups, which appear to represent two different blots, suggesting digital splicing
- Similarities between Figure 6A OA and miR26a inhibitors+BMS-34551 panels with rotation, as well as Figure 6B OA and miR26a inhibitors+BMS-34551 panels with rotation, and Figure 6D BMS-345541 and miR-26a inhibitors+BMS-34551 panels

The authors were initially contacted with regards to the reader's concerns and did not respond, but the author Zhi Zhao has since contacted the journal requesting a retraction of this article, stating the authors have been unable to replicate the results. The other authors have not responded. The Editor-in-Chief and Editorial Board agree with the retraction.

